# Markers of Deception in Italian Speech

**DOI:** 10.3389/fpsyg.2012.00453

**Published:** 2012-10-30

**Authors:** Katelyn Spence, Gina Villar, Joanne Arciuli

**Affiliations:** ^1^Faculty of Health Sciences, University of SydneySydney, NSW, Australia

**Keywords:** deception, lying, linguistic markers of deception, cross-linguistic, Italian

## Abstract

Lying is a universal activity and the detection of lying a universal concern. Presently, there is great interest in determining objective measures of deception. The examination of speech, in particular, holds promise in this regard; yet, most of what we know about the relationship between speech and lying is based on the assessment of English speaking participants. Few studies have examined indicators of deception in languages other than English. The world’s languages differ in significant ways, and cross-linguistic studies of deceptive communications are a research imperative. Here we review some of these differences amongst the world’s languages, and provide an overview of a number of recent studies demonstrating that cross-linguistic research is a worthwhile endeavor. In addition, we report the results of an empirical investigation of pitch, response latency, and speech rate as cues to deception in Italian speech. True and false opinions were elicited in an audio-taped interview. A within-subjects analysis revealed no significant difference between the average pitch of the two conditions; however, speech rate was significantly slower, while response latency was longer, during deception compared with truth-telling. We explore the implications of these findings and propose directions for future research, with the aim of expanding the cross-linguistic branch of research on markers of deception.

## Introduction

Deception can take many forms. Whether it be exaggeration, equivocation, concealment, or an outright lie, deception is a deliberate act that originates with the intent to mislead others (DePaulo et al., 2003). It has been suggested that people lie on average once a day during routine social interactions (DePaulo et al., 1996). Given that we come into contact with lies every day, it is perhaps surprising to discover that many people find it difficult to detect deception. A meta-analysis of 206 studies revealed that humans perform near chance (54%) when making veracity judgments (Bond and DePaulo, 2006). However, most studies involve the elicitation of lies through low-stakes, laboratory-based paradigms and it should be acknowledged that some professional lie-catchers are capable of accuracy rates that are significantly higher than this (Frank and Svetieva, 2012), particularly when they are asked to make veracity judgments in real-life, high-stakes circumstances (Mann et al., 2004). One explanation for poor deception detection performance is that, generally, people hold inaccurate beliefs about what constitutes a reliable indicator of deception (Vrij, 2000). Examination of participants from 75 countries and 43 languages demonstrated that inaccurate beliefs about lie detection are common (Global Deception Research Team, 2006). For example, many people believe that gaze aversion indicates deception (Vrij et al., 2006), a conviction that can compromise lie detection accuracy (Forrest et al., 2004). More recently, it has been suggested that difficulties in lie detection stem from weak associations between cues and deception, rather than people’s reliance on inaccurate beliefs about reliable indicators of deception (Hartwig and Bond, 2011).

Regardless of the underlying cause, the mediocre deception detection rates of the average human observer have impelled the search for objective indicators of lying. Traditionally, objective analyses of lying behavior have been grouped into psychophysiological measures (e.g., heart rate and skin conductivity), “verbal” cues (e.g., the presence of emotive words), and other cues. The latter have sometimes included visual behaviors (e.g., gestures, facial expressions) and what have been referred to as “vocal” or “paraverbal” indices (e.g., pitch and speech rate, see Sporer and Schwandt, 2006, for a review). Here, we have chosen to adopt the term “linguistic” cues, which includes any behavior that is directly associated with oral or written communication. From this perspective, linguistic indicators of lying include the content of both spoken utterances and written communications (e.g., lexical content such as parts-of-speech), along with measures that reflect the way that communication is being delivered (e.g., the analysis of pitch in the case of spoken utterances). A now sizeable body of research has investigated the utility of linguistic cues to deception; however, this research has focused primarily on speakers of English. Lying is a universal activity; hence, it is important to examine linguistic markers of deception beyond English. In the current study, we provide an overview of cross-linguistic research on markers of deception and present empirical data on three potential markers of deception in Italian speech: pitch, response latency, and speech rate.

### Theories of deception

A number of theories have been proposed to explain behavioral differences between deception and truth-telling, including the Four-Factor Model, Interpersonal Deception Theory, the Motivation Impairment Effect, and the Self-Presentational Perspective (for a review, see DePaulo et al., 2003). One of the most influential of these theories is Zuckerman et al.’s (1981a) Four-Factor Model. This model attempts to explain cues to deception in terms of four psychological processes that may occur during lying compared to truth-telling, specifically: *generalized arousal*, in response to *increased emotion* (fear, guilt, or excitement at deceiving), *cognitive load* (presumably it requires concerted cognitive effort to fabricate a coherent, plausible, consistent account, and maintain a deception), and *attempted control* (deliberate self-regulatory strategies to suppress any leakage of cues). Too much control could result in telling behaviors such as a reduction in emotional expressiveness or reduced hand movement. Alternatively, it may be difficult for deceivers to control all communication channels simultaneously. For example, a deceiver may focus primarily on controlling their facial expression but exert less control over other behaviors.

There is some evidence for Zuckerman et al.’s (1981a) Four-Factor Model to suggest that people do experience one or more of these psychological processes more frequently during deceptive than truthful behavior (e.g., Dionisio et al., 2001; Walczyk et al., 2003; Caso et al., 2005; Gombos, 2006). However, which of these processes will dominate under what circumstances, and which cues to deception are indicative of each of these processes is still being debated in the literature (DePaulo et al., 2003; Caso et al., 2005; Gombos, 2006). While there is debate over the extent to which such processes are under the control of the deceiver, there is general agreement that some cues to deception are non-strategic and frequently outside the deceiver’s awareness (DePaulo et al., 2003). It is feasible that some acoustic behaviors, such as pitch and speech rate, might be less vulnerable to behavioral control than other linguistic markers of lying (Villar et al., in press). Vocal pitch, for example, may be more difficult to manipulate when it represents an autonomic response to strong emotion, such as the anxiety an individual may experience while lying (Zuckerman et al., 1981a).

### Markers of deception

The ongoing challenge in lie detection is that there is no single behavior that occurs in all people in every situation and is exclusively related to deceptive behavior (DePaulo et al., 2003). However, some behaviors appear to be more reliable than others. In their meta-analysis, DePaulo and colleagues reviewed 116 studies and coded 158 different cues to deception. These included facial expressions, physical behaviors, and language-related measures (including acoustic measurements). Significant relationships were found between deception and behavioral cues in each of these categories. The results led to the conclusion that liars are “less forthcoming, less compelling, more negative, more tense, and suspiciously bereft of ordinary imperfections and unusual details” (p. 104). Sporer and Schwandt (2006) conducted a meta-analysis of 41 studies that focused on nine cues: speech rate, response latency, message duration, number of words, filled and unfilled pauses, repetitions, speech errors, and pitch. Results indicated that of these cues only pitch (*d* = 0.268) and response latency (*d* = 0.177) were reliably associated with deception, with both showing increases during lying compared to truth-telling.

### Cross-linguistic research

The world’s languages differ in many ways, and it follows that there might be differences in the extent to which the cues which have been previously identified as viable markers of lying in English can be applied across languages. Take the case of grammatical category. A decrease in personal pronoun use has been observed in lying compared to truthful speech for English speaking participants. However, personal pronoun use is overt in English most of the time, so this begs the question: does the deception detection utility of pronoun use extend to null personal pronoun languages, such as Italian and Spanish, where pronoun use is overt only 20–30% of the time (Serratrice, 2005), or to languages such as Japanese which uses considerably fewer pronouns in general than Indo-European languages (Shibatani, 1990)? Likewise, an increase in adjective and adverb use has been observed in lying compared to truthful speech for English speaking participants (Zhou et al., 2004). Yet, not all languages use the same grammatical categories; for instance, Russian has no phrasal verbs (Mudraya et al., 2008), and Polish has no articles (Wierzbicka, 1985). Silent pause duration is another linguistic variable thought to be an indicator of deception in English (Mann et al., 2002); yet, pause duration differs among languages. For example, native speakers of Russian use longer pauses during informal monologs than do native speakers of English (Riazantseva, 2009), while the latter demonstrate shorter silent pauses in read speech than do native speakers of Italian (Campione and Véronis, 2002). The extent to which these differing characteristics are culturally derived is open to debate. Nonetheless, such differences underscore the importance of investigating cues to deception in a range of speakers including but not restricted to English speaking participants.

Previous research on linguistic indicators of deception includes a substantial body of work devoted to two language assessment tools, namely, Criteria-Based Content Analysis (CBCA; Steller and Köehnken, 1989) and Reality Monitoring (RM; Johnson and Raye, 1981). These tools have been successfully used with adult speakers of German, Swedish, Dutch, French, Spanish, and English for the identification of true versus fabricated narratives (Ruby and Brigham, 1997; Vrij et al., 2004; Blandón-Gitlin et al., 2009). Another credibility assessment technique, which is partly derived from CBCA and RM, is Assessment Criteria Indicative of Deception (ACID; Colwell et al., 2007). This tool has been applied to the credibility assessment of Arabic speakers; although, the analysis was performed on the English translation of their oral statements, as opposed to assessing the Arabic utterances directly (Colwell et al., manuscript in progress, cited in Suckle-Nelson et al., 2010). When implemented by trained assessors, each of these techniques can discriminate deceptive from truthful narratives at rates that are higher than chance; however they are labour-intensive and dependent upon contextual clues to veracity (Masip et al., 2005; Vrij, 2005). Evaluating the utility of other markers of lying, that can be measured independent of the judgment of a trained observer, is a worthwhile endeavor. To this end, computerized text analysis programs, such as Linguistic Inquiry Word Count (LIWC; Pennebaker et al., 2007) have been applied to the identification of deceptive text and transcribed verbal utterances in languages other than English, including Spanish, Dutch, Italian, and German (e.g., Schelleman-Offermans and Merckelbach, 2010; Fornaciari and Poesio, 2011; Almela et al., 2012; Hauch et al., 2012; Masip et al., 2012; Sporer, 2012).

Some deception studies do not specify the language in which the lies are elicited, and we are left to deduce the target language from the location of the laboratory in which the research was conducted. Of those that do specify a language other than English, there appear to be few studies which have examined linguistic markers of deception without the input of a trained assessor (e.g., Anolli and Ciceri, 1997; Anolli et al., 2003; Zhou and Sung, 2008; Schelleman-Offermans and Merckelbach, 2010). Some of the variables that were revealed to be viable markers of deception in English have shown mixed results in studies of other languages. For example, Zhou and Sung (2008) examined the computer-mediated communications of Chinese players engaged in a so-called Mafia Game. Results revealed that, consistent with some studies of English speakers, the use of third person pronouns increased during deception. Inconsistent with findings from some studies of English speakers, there were no significant differences between the proportional use of first person pronouns in the deceivers’ versus truth-tellers’ messages; however, one limitation of the study reported by Zhou and Sung (2008) was the use of a between-participants design. In a within-subjects design, Schelleman-Offermans and Merckelbach (2010) examined the presence of self-references in the true compared to the fabricated written stories of Dutch speakers. Among other findings, the results showed no significant differences between the presence of self-references in participants’ true versus deceptive narratives. While there are methodological differences that may account for the dissimilarities between these findings and those of studies with English speakers, it is possible that some cues which have shown promise in English are not as useful in other languages.

Notably, most studies in languages other than English have examined lying in computer-mediated communication (e.g., Zhou and Sung, 2008), through the written modality (e.g., Schelleman-Offermans and Merckelbach, 2010) or via language analysis of transcribed speech (e.g., CBCA, RM, and ACID). Only a handful of studies (e.g., Anolli and Ciceri, 1997; Anolli et al., 2003) have examined the cross-linguistic utility of acoustically quantifiable markers of deceptive speech. Pitch, response latency, and speech rate are three such variables which have received some attention in studies of English and non-English speaking participants.

#### Pitch

Pitch refers to our perceptions of how “low” or “high” a voice sounds. The acoustic correlate of pitch is fundamental frequency (*F*_0_), which is a measure of the frequency of vibrations of the vocal tract during speech production. Automated acoustic analysis programs, such as Praat (Boersma and Weenink, 2011), can be used to measure *F*_0_. Adult males produce an average *F*_0_ between 100 and 150 Hz, while adult females’ *F*_0_ tends to be higher with an average between 175 and 250 Hz (Baken and Orlikoff, 2000). The effects of pitch have been noted in situations that vary in terms of emotional involvement. For example, pitch has been shown to increase in situations that evoke strong emotions such as viewing pictures of burn victims (Ekman et al., 1991), and discussing personal beliefs and future plans (Streeter et al., 1977).

While some studies have reported no pitch differences between liars and truth-tellers (Buller and Aune, 1987; Bond et al., 1990; Vrij and Winkel, 1991; Fiedler and Walka, 1993), the findings of two seminal meta-analyses provide support for an overall increase in average pitch across multi-word deceptive compared to truthful utterances (DePaulo et al., 2003; Sporer and Schwandt, 2006). In addition to studies of average pitch, the deception literature contains examinations of pitch variability (measured as standard deviation of *F*_0_). Various studies have found that there is a significantly greater variation in pitch during deceptive speech compared to truthful speech.

An increase in average pitch, and pitch variability during lying, might be due to an increase in arousal during lying that leads to physiological responses in the body that are difficult to control (Zuckerman et al., 1981a; Sporer and Schwandt, 2006). Heightened emotion, such as the anxiety that is commonly experienced during deception, is thought to intensify tension in the vocal tract, which is responsible for the increase in pitch that accompanies lying. Of relevance to the current study, increases in average pitch and pitch variability have been observed during lying compared to truth-telling in the speech of 31 male Italian undergraduate students (Anolli and Ciceri, 1997). An examination of pitch in deceptive Italian speech, using a sample that includes female participants and older participants (as opposed to a sample comprised entirely of college students), is required.

#### Response latency

Response latency is the amount of time taken to respond to a question or statement. Several studies have used this definition to measure response latency in relation to deception (e.g., Rockwell et al., 1997b; Feeley and deTurck, 1998; Vrij et al., 2000). Some have reported no difference (Buller et al., 1989) or a decrease (O’Hair et al., 1981; Dulaney, 1982) in response latency in deceptive compared with truthful speech. It has been suggested that decreases in response latency during lying might be a result of the speakers’ beliefs that faster responses are associated with a more credible impression (Dulaney, 1982; Buller et al., 1989).

As revealed by the results of Sporer and Schwandt’s (2006) meta-analysis, other studies have found that response latency increases during deception compared to truth-telling (e.g., Harrison et al., 1978; deTurck and Miller, 1985; Feeley and deTurck, 1998; Vrij et al., 2000). An increase in response latency has been attributed to the increased cognitive load experienced by a deceiver (Vrij et al., 2000; Sporer and Schwandt, 2006). At the time of writing, we are unaware of any studies which have examined response latency in the speech of Italian speakers during lying compared to truth-telling.

#### Speech rate

Speech rate refers to the speed with which someone speaks, and can be measured in a variety of ways. Measures of the number of words and syllables, divided by the acoustic length of the utterance (in seconds) are the most common in the deception literature (DePaulo et al., 1982; Riggio and Friedman, 1983; Buller and Aune, 1992; Rockwell et al., 1997a; Feeley and deTurck, 1998; Vrij et al., 2000). Significant variations in speech rate between speakers within the same language have been reported (Ramus, 2002); therefore, it is difficult to refer to an average speech rate for adult speakers. However, the average articulation rate of spontaneous Italian speech has been estimated at 4.9 syllables and 3.4 words per second (Caldognetto et al., 1997). Cross-linguistic investigations have found that speech rate can also vary between languages. For example, German speakers articulate significantly faster than Italian speakers (Russo and Barry, 2008).

The relationship between speech rate and deception is equivocal in the deception literature. In several studies, significant decreases in speech rate during deceptive versus truthful utterances have been observed (Fiedler and Walka, 1993; Ebesu and Miller, 1994; Rockwell et al., 1997b; Vrij et al., 2000; Vrij and Mann, 2001; Vrij et al., 2008), while non-significant decreases have been observed in some (Mehrabian, 1971; Hocking and Leathers, 1980; Feeley and deTurck, 1998), including one study of 31 male Italian speakers (Anolli and Ciceri, 1997). Decreases in speech rate during lying have been attributed to the increase in cognitive load that is thought to accompany lying (Vrij et al., 2008). Significant increases in speech rate during deception have been observed in other studies (Mehrabian, 1971; Klaver et al., 2007). It is possible that methodological differences, particularly in the extent to which participants are cognitively challenged by the experimental task, might account for the different outcomes that have been observed across studies. For example, when given little time for planning, liars speak more slowly than truth-tellers; however, the opposite has been observed when liars are given opportunities to prepare their lie (Sporer and Schwandt, 2006). Participants in the current study were given no preparation time prior to the elicitation of their deceptive response, in order to increase the cognitive challenges of the task.

### The current study

In summary, deceivers are prone to experiencing (consciously or otherwise) heightened emotion, increased cognitive effort, and attempts at behaviour control (DePaulo et al., 2003; Vrij, 2008). Deceivers may experience the same psychological processes regardless of their background; however, these processes may have different behavioral manifestations depending upon linguistic and/or cultural context. Previous research has investigated the utility of a number of cues to deception. Of these potential deception markers, pitch, response latency, and speech rate were selected for the current study.

In line with previous research conducted with English speakers, and one study of male Italian speakers (Anolli and Ciceri, 1997), it was hypothesized that pitch would be higher in the deceptive speech compared to the truthful speech of Italian speakers. Additionally, it was hypothesized that response latency would be longer in deceptive speech. Due to inconsistencies in the findings of previous studies, the direction and significance of differences in speech rate during deception versus truth-telling was an open empirical question. In light of individual variability amongst participants in terms of their personal speaking style, including differences in pitch, response latency, and speech rate, we employed a within-participants design.

## Materials and Methods

### Participants

Nineteen native speakers of Italian (12 females and 7 males) with a mean age of 56.1 years (*SE* = 3.36) participated in this study. They were recruited in Sydney, Australia, through a variety of methods including word of mouth, advertisements in a local Italian newspaper, and flyers distributed at Italian community organizations. All participants were born and educated in Italy.

### Procedure

Recruitment materials described the study as an investigation of communication skills relating to social issues, in order to avoid attracting participants who considered themselves to be particularly good liars, or those who considered themselves to be poor liars and were hoping to improve their abilities. The same researcher, who was a native speaker of Italian, conducted all of the individual testing sessions in Italian, which took approximately 30 minutes each. All materials and consent forms were provided in Italian.

We employed the well-established false opinion paradigm based on the procedure described by Frank and Ekman (2004) which has been used in a variety of laboratory-based studies of deception (Newman et al., 2003; Arciuli et al., 2010; Villar et al., in press). Participants completed a questionnaire to determine their opinions on various social issues. These social issues are listed in Table 1.

**Table 1:** **Topics addressed in social issues questionnaire (translated into English here)**.

Topic	Description
Smoking in public	Should smoking be banned in all enclosed public spaces?
Capital punishment	Should the death penalty be reintroduced in Italy/Australia?
Legalization of marijuana	Should marijuana be legalized for public use?
Legalization of abortion	Should abortion be legal in Australia/Italy?
Same-sex marriage	Should same-sex couples be allowed to marry?
Sex offender registry	Should the identity and location of sex-offenders be made public on the internet?
Church versus state	Should the church be allowed to intervene in political decisions?
Blood alcohol limit	Should the legal blood alcohol limit for driving be lowered from 0.05 to 0.02?

Participants were asked to rate the extent to which they agreed or disagreed with each social issue (*“1”* = *completely disagree, “7”* = *completely agree)* as well as the strength of their feelings about the issue (*“1”* = *No feelings, “7”* = *Very strong feelings*). Two issues were then selected for each participant, one about which they would lie, and one about which they would tell the truth. Topics where participants reported strong opinions and strong feelings were chosen. The mean absolute difference of *opinion* ratings from the midpoint of 4 (i.e., mean strength of agreement or disagreement measured as the distance of the value from zero: 1 and 7 become 3, 2 and 6 become 2, and 3 and 5 become 1) were 2.84 (*SE* = 0.12) for the truthful target topics and 2.74 (*SE* = 0.15) for the untruthful target topics. One-sample *t*-tests revealed significant differences between zero and the mean absolute difference of *opinion* ratings for the strength of agreement with the truthful topics [*t*(18) = 24.705, *p* < 0.0001] and the untruthful topics [*t*(18) = 18.258, *p* < 0.0001]. A paired samples *t*-test revealed no significant difference between these mean of 2.84 and 2.74 [*t*(18) = 0.622, *p* = 0.542]. The mean absolute difference of *feelings* ratings from the midpoint were 2.63 (*SE* = 0.18) for the truthful target topics and 2.26 (*SE* = 0.25) for the untruthful target topics. One-sample *t*-tests revealed significant differences between zero and the mean absolute difference of ratings of the strength of participants’ *feelings* toward the truthful topics [*t*(18) = 15.076, *p* < 0.0001] and the untruthful topics [*t*(18) = 8.988, *p* < 0.0001]. A paired samples *t*-test revealed no significant difference between these mean of 2.63 and 2.26 [*t*(18) = 1.235, *p* = 0.233]. Hence, participants’ opinions and feelings were (i) sufficiently strong and (ii) equivalent across true and false topics.

Participants were randomly assigned to lie about one of the designated issues and tell the truth about the other. The order of topics was counterbalanced such that half the participants started the interview with a lie and half with the truth. To determine the effect of topic on each of the target variables, one-way ANOVA were conducted. Results revealed that there was no significant effect of topic on pitch [*F*(7, 11) = 1.947, *p* = 0.155], response latency [*F*(7,11) = 0.857, *p* = 0.566], or speech rate [*F*(7,11) = 2.362, *p* = 0.098].

Participants were instructed to provide an honest account of their true opinion of the topic designated for the truthful condition, along with a false representation of their true opinion for the topic designated for the deceptive condition. Participants were told that the interviewer would not know whether they were lying or telling the truth and that they should aim to convince him of their credibility in each of the interviews. Participants were not given any planning time during which to prepare their false or true opinion. The topic was read aloud to the participant who was then asked to state whether they agreed or disagreed and explain why. This was then followed up with a question enquiring whether they were telling the truth. At the conclusion of the interview participants were debriefed and thanked for their cooperation. Interviews were recorded using a Sony Digital Voice Recorder, which has a frequency response of between 80 and 20,000 Hz. All audio files were stored in uncompressed linear PCM (.wav) format for later analysis.

### Data preparation and analysis

A native speaker of Italian performed a verbatim Italian transcription of all the interviews. Praat software (Boersma and Weenink, 2011) was used to measure pitch, response latency, and length of utterance (used to calculate speech rate) in each of the audio recordings. In line with Praat software instructions, the speech samples were analyzed using a pitch range of 75–500 Hz for females, and 75–300 Hz for males. Response latency was determined by measuring the time lapse from the end of the first question asked by the interviewer and the start of the participants’ response in milliseconds. Duration of response latency was measured via visual examination of the wave form. The portion of the wave form that represented the response latency was magnified, permitting accurate selection and measurement of the latency duration in milliseconds (ms). Recent research suggests that interjections such as “erm” and “um” constitute lexical terms (Arciuli et al., 2010; Villar et al., 2012), and so these were included in the total word count in each transcription. Speech rate was calculated by dividing the total number of words in the utterance, by the acoustic length (measured in seconds).

## Results

### Word count

The average number of words produced in the deceptive speech condition was 189.63 (*SE* = 17.21), while the average number of words in the truthful speech condition was 218.84 (*SE* = 20.27). A paired samples *t*-test showed no significant difference between these means [*t*(18) = 1.162, *p* = 0.260, two-tailed].

### Acoustic duration

The average acoustic duration of the responses in the deceptive speech condition was 100.20 s (*SE* = 9.46). The average duration of the responses in the truthful speech condition was 104.03 s (*SE* = 9.70). A paired samples *t*-test revealed no significant differences between these means [*t*(18) = 0.322, *p* = 0.751, two-tailed].

In order to assess the reliability of the measure of duration of utterance, a second rater measured this variable for just over 50% of the 38 observations (*n* = 20). The inter-rater reliability coefficient was significant (*r* = 0.927, *p* < 0.001), indicating a high consistency between the measurements of acoustic duration that were recorded by the two raters.

### Pitch

The average pitch of participants in the deceptive condition was 160.88 Hz (*SE* = 7.73). The average pitch in the truthful condition was very similar at 160.67 Hz (*SE* = 7.19). A paired samples *t*-test revealed no significant difference between the average pitch across conditions [*t*(18) = 0.093, *p* = 0.927, two-tailed] and the effect size was small (*d* = 0.006). In view of the differences in pitch between male and female speakers, additional analyses were performed. The average pitch of female speakers was 178.14 Hz (*SE* = 6.24) during their truthful utterances and 180.21 Hz (*SE* = 6.50) during their deceptive utterances. The average male pitch was 130.74 Hz (*SE* = 7.87) during their truthful utterances and 127.76 Hz (*SE* = 8.04) during their deceptive utterances. As expected, an analysis of gender effects on pitch production, a 2 (veracity: lying versus truth-telling) × 2 (gender: male versus female) mixed ANOVA revealed a significant main effect of gender [*F*(1,17) = 24.632, *p* < 0.0001, partial η^2^ = 0.592]. However, there was no significant main effect of veracity [*F*(1,17) = 0.037, *p* = 0.850, partial η^2^ = 0.002] and no interaction between gender and veracity [*F*(1,50) = 1.147, *p* = 0.299, partial η^2^ = 0.063].

Further analyses were performed to determine variability in pitch (measured as the standard deviation of *F*_0_, in Hz) in each condition. A paired samples *t*-test revealed no significant difference between pitch variability in the true (*M* = 56.12, *SE* = 5.67) compared to the lying (*M* = 57.60, *SE* = 5.91) condition [*t*(18) = 0.344, *p* = 0.735, two-tailed, *d* = 0.06]. Using a median split analysis, the variable of average *F*_0_ was dichotomized into groups (variability: low/high) for each condition (truthful/deceptive). An independent samples *t*-test revealed no significant difference between the average *F*_0_ of the truthful (*M* = 163.30, *SE* = 7.30) compared to the deceptive (*M* = 173.70, *SE* = 6.30) conditions for the low variability group [*t*(17) = 1.066, *p* = 0.301, two-tailed], even in view of a moderate effect size (*d* = 0.49). Similarly, for the high variability group, there were no significant differences between the average *F*_0_ of the truthful (*M* = 157.75, *SE* = 12.30) and the deceptive (*M* = 149.35, *SE* = 149.35) conditions [*t*(17) = 0.454, *p* = 0.655, two-tailed]. The effect size was small (*d* = 0.20).

### Response latency

A Kolmogorov–Smirnov test of the entire data-set revealed that the distribution of scores for response latency in the truthful speech condition, *D*(19) = 0.350, *p* < 0.001, and the deceptive speech condition, *D*(19) = 0.378, *p* < 0.001, were both significantly non-normal. Consequently, the data were analyzed using a non-parametric alternative to a paired samples *t*-test: the Wilcoxon signed-rank test. Results showed that response latency (in ms) was longer in the deceptive speech condition (Mdn = 1200.77) than in the truthful speech condition (Mdn = 775.26). This difference was significant, *T* = 9.50, *p* = 0.02, and the effect size was large (*r* = −0.51).

A second rater measured response latency in just over 50% of the 38 observations (*n* = 20). The inter-rater reliability coefficient was significant (*r* = 0.998, *p* < 0.001), indicating a high consistency between the measurements of response latency that were recorded by the two raters.

### Speech rate

The average speech rate (in words per second) was slower in the deceptive speech condition (*M* = 1.95, *SE* = 0.08) compared to the truthful speech condition (*M* = 2.10, *SE* = 0.07). A paired samples *t*-test revealed a significant difference between the means [*t*(18) = 2.454, *p* = 0.025, two-tailed]. The effect size was medium (*d* = 0.447).

## Discussion

Here we examined whether pitch, response latency, and speech rate are helpful in distinguishing between deceptive and truthful communications in Italian. Our hypothesis that pitch would be higher in the deceptive speech condition was not supported. As hypothesized, we found that response latency was significantly longer in the deceptive speech condition compared to the truthful speech condition. It was an open empirical question as to whether participants’ speech rate would differ during lying compared to truth-telling. The data revealed a significant difference between the average speech rate for the two conditions: speech rate was significantly slower in the deceptive versus the truthful speech condition. The lies in the present study were, on average, of a relatively short duration (around 100 s); yet, they were of a sufficient length to enable the detection of significant changes in response latency and speech rate during lying compared to truth-telling.

### Pitch

It has been documented that increased pitch is one of the cues that people associate with deceptive speech (Zuckerman et al., 1981b; Vrij and Semin, 1996; Anderson et al., 1999; Lakhani and Taylor, 2003; Colwell et al., 2006). Speakers sometimes employ counter-measures to appear more believable when they lie (Sip et al., 2008). Consequently, it is possible that some of the subjects in our study strategically managed their vocal pitch in an attempt to appear more credible. However, a recent study found that those individuals who believed that pitch increases during deception, demonstrated a significantly higher pitch during their own deceptive utterances (Villar et al., in press). Thus, it is unlikely that attempts at behavioral control can explain the findings in the current study.

The pitch values we observed are in line with previous reports of pitch in adult females and males (Villar et al., in press). Additional analyses were conducted in order to determine whether differences in pitch across females and males may have influenced the mean pitch results. There was no main effect for veracity, nor a significant interaction between gender and veracity. Therefore, it is unlikely that gender had a systematic impact on our mean pitch results.

In addition to measuring mean pitch, variability in pitch is another frequently used measure in voice research (Neil et al., 2003). Deception research, also, has looked at the effects of lying on pitch variation and found greater pitch variation in deceptive speech compared to truthful speech (Anolli and Ciceri, 1997; Rockwell et al., 1997a). Our analyses indicated no significant difference in pitch variability across the truthful versus deceptive conditions, nor was there a significant difference in the average *F*_0_ between the truthful and deceptive conditions for either the high variability group or the low variability group. Thus, regardless of whether a speaker’s pitch variability was high or low there was no difference in the average vocal pitch of their truthful compared to their deceptive speech.

Sporer and Schwandt’s (2006) meta-analysis found that pitch was significantly higher during lying when participants lied about “facts and feelings,” as opposed to “facts only.” The explanation offered for this finding is that, in the absence of increased emotional arousal, pitch remains the same during lying compared to truth-telling. It is possible that we did not observe the expected increases in pitch during lying because of the paradigm we used to elicit the lies. Perhaps, despite our attempts to elicit topics about which participants felt strongly (see Materials and Methods), the topics were not sufficiently arousing to be accompanied by pitch changes. However, this explanation seems problematic given that our paradigm was successful in eliciting differences in response latency and response rate in lying versus truthful Italian speech.

While all the participants in our study were native speakers of Italian who were born and educated in Italy, they were also speakers of English who were visiting or residing in Australia. It is possible that the bilingual status of the participants influenced their speech. However, we think it unlikely that bilingual status would have a systematic impact upon truth-telling and lies such that exposure to English as a second language would result in a lack of pitch differences in native Italian speech. The speech of bilinguals has been shown to incorporate phonological and prosodic features from both languages (Jusczyk, 1997). Thus, having English as a second language might *increase* the likelihood that Italian speakers would speak in a higher pitch during lying compared to truth-telling, in the same way that English speakers appear to. However, this was not the case in the current study. Anolli and Ciceri (1997) reported significant differences in the mean, range, and variability in pitch of the deceptive versus truthful utterances of their male Italian participants. Their participants were younger than ours (*M* = 24.4 years versus *M* = 56.1 years). Perhaps there are age-related differences in pitch during lying compared to truth-telling that could account for the differences between our findings and those of Anolli and Ciceri.

Lastly, it is feasible that there are differences between languages in the efficacy of pitch as an indicator of deception, which are culturally, as opposed to linguistically determined. For example, Van Bezooijen (1995) showed that Japanese women produce a higher pitch on average than Dutch women, and suggested that these differences reflect the characteristics that are perceived to be desirable in women in each culture (i.e., a preference for high pitch in Japanese women, and low to medium pitch in women from the Netherlands). Future studies might consider the socio-cultural factors that could influence the viability of pitch as a marker of deception in languages other than English.

Further research is required in order to explore these possibilities.

### Response latency

The response latency results are in line with the findings of Sporer and Schwandt’s (2006) meta-analysis. Of note, we observed a large effect size concerning response latency. As explained by the four-factor theory (Zuckerman et al., 1981a), a longer response latency during deception may be due to the increased cognitive load associated with lying which can lead to “leakage” of certain behaviors (Vrij et al., 2000; Sporer and Schwandt, 2006). Sporer and Schwandt (2006) proposed that the increased cognitive load experienced during deception is due to increased demands on working memory. In other words, when a pre-existing schema or script is not available, which is often the case during lying, the formulation of novel ideas is required. This increases the load on working memory, leaving less capacity available for speech production, which can lead to increased latencies. Short planning times for lie formulation have been associated with longer latencies (Sporer and Schwandt, 2007), and it is possible that the low levels of preparation time in the current study contributed to the efficacy of this variable.

### Speech rate

Previous studies of speech rate during lying compared to truth-telling have produced conflicting results. Our findings are consistent with those studies which have found that speech rate is significantly slower during lying compared to truth-telling (Fiedler and Walka, 1993; Ebesu and Miller, 1994; Rockwell et al., 1997b; Vrij et al., 2000; Vrij and Mann, 2001; Vrij et al., 2008). Once again, the increases in cognitive load that are thought to accompany lying might reduce the cognitive capacity available for other activities, such as speech production (Sporer and Schwandt, 2006). One consequence of this might be the slower speech that we have observed here during lying. Notably, our findings are in the same direction as those of Anolli and Ciceri (1997) who found a decrease (albeit a non-significant one) in speech rate during lying compared to truth-telling for their 31 male Italian speakers. It is worth noting the different methodologies that were utilized: Anolli and Circeri’s participants described a black and white picture, while participants in the current study described opinions of social topics. It could be argued that the latter involves a more emotive and cognitively demanding task (but that explanation becomes a little problematic when interpreting discrepant results between the two studies concerning pitch).

Future studies might consider the cross-linguistic utility of speech rate as a marker of lying in languages other than English and Italian. It may be that measures of words per second are not appropriate for all languages. For instance, in languages such as Japanese and Filipino, where the morphology is highly agglutinative, a more appropriate measure of speech rate might be the number of morphemes per second.

## Conclusion

This research investigated the effects of veracity on pitch, speech rate, and response latency in the speech of native speakers of Italian. Each of these variables has been linked to deception in the speech of native speakers of English. Our findings revealed that response latency and speech rate are associated with deception in the speech of native speakers of Italian in the same way that they are for English speakers. No relationship was found between pitch and lying in the present study. Additional studies are required to determine whether pitch is a reliable marker of lying in languages other than English. Further investigations of the extent to which differences in deceptive communications across languages are linguistically, as opposed to culturally derived, are also required. In our view, a systematic analysis of the utility of a range of linguistic variables in cross-linguistic and cross-cultural contexts would be invaluable for deception research. Another very interesting avenue for research is the comparison of linguistic cues to deception in monolingual and bilingual speakers (including comparison of simultaneous bilinguals versus those that acquired a second language after acquiring their first). It would also be valuable to assess lying versus truth across multiple languages in a within-subjects design to see if cues to deception are used by the same multilingual speakers regardless of the language they are speaking. We hope that the current study encourages expansion of this line of deception research. It remains to be seen whether a unique pattern of cues to deception will emerge for each language or whether we will discover that there are some markers of lying that are common across languages.

## Conflict of Interest Statement

The authors declare that the research was conducted in the absence of any commercial or financial relationships that could be construed as a potential conflict of interest.
